# Identifying the human olfactory and chemosignaling neural networks using event related fMRI and graph theory

**DOI:** 10.1038/s41598-025-96355-2

**Published:** 2025-04-08

**Authors:** Saideh Ferdowsi, Tom Foulsham, Alireza Rahmani, Dimitri Ognibene, Luca Citi, Wen Li

**Affiliations:** 1 School of Mathematics, Statistics and Actuarial Science, University of Essex, Colchester, UK; 2 Department of Psychology, University of Essex, Colchester, UK; 3 Department of Psychology, University of Milano-Bicocca, Milan, Italy; 4 School of Computer Science and Electronic Engineering, University of Essex, Colchester, UK; 5 Louis A. Faillace, MD, Department of Psychiatry and Behavioral Sciences, University of Texas Health Science Center, Houston, TX USA; 6 Islamic Azad University, Tehran, Iran

**Keywords:** Neuroscience, Psychology, Biomarkers, Neurology, Mathematics and computing

## Abstract

This study aims to characterize and compare the functional neural networks associated with different olfactory stimuli, including air, non-social odours, and human body odours. We introduce a novel processing pipeline based on event-related functional magnetic resonance imaging (fMRI) and graph theory for network identification. To ensure the stability and small worldness of the characterized networks, we conduct statistical validations, network modularity assessments, and robustness measurement against local attacks. The key hypothesis is that human body odours (so-called social odours) and non-social odours engage distinct neural networks, particularly in regions responsible for social processing. We found that the posterior medial orbitofrontal cortex (pmOFC) and fusiform face area (FFA) demonstrate stronger centrality in the body odour network than the non-social odour and air networks. This observation supports the idea that social and olfactory information are integrated in the body odour network. Additionally, the anterior insula (INSa), posterior piriform cortex (PPC), and amygdala (AMY) exhibit high influence in air and odour networks by achieving higher centrality indices and playing a major role in improving the global efficiency. These findings offer impactful insight into how air, non-social, and social odours recruit distinct neural circuits, reinforcing the role of olfaction in human social behavior.

## Introduction

The human olfactory system plays a fundamental role in daily life influencing both physiological and behavioural states^1–3^. Researchers believe that this role is driven by a distributed network encompassing sensory, limbic, and higher-order cognitive regions^4–6^. Although neuroimaging techniques have significantly improved the study of brain networks, identifying the olfactory-evoked network remains challenging due to two key factors. First, olfactory processing involves complex structural and functional connections among cortical and subcortical brain regions making precise mapping complicated^4–7^. Second, as evidenced by the researches, different categories of olfactory stimuli such as air, social odours (e.g. human body odours mainly fund in sweat) and non-social odours (e.g. environmental scents) recruit distinct brain networks, further complicating their characterization^2,8,9^. To advance our understanding of the olfactory processing network, this study aims to characterize and compare the neural circuits associated with three categories of olfactory stimuli including social odours, non-social odours, and air using event-related fMRI. Studies have shown that human body odours operate as social signals transmitting socially relevant information using chemical compounds^3,4^. The ability to convey characteristics such as age and gender through non-experimentally manipulated body odours indicates the strength of this olfactory cue in activating the social brain network^10,11^. For this reason, body odour is recognized as a chemosigna because it acts as a chemical signal that conveys information about an individual to others. This study also uses the term chemosignal for human body odours.

### Neural network evoked by air

In a comprehensive study performed by Arnold et al.^5^, the intrinsic human olfactory network was assessed using two fMRI datasets and graph theory. The fMRI datasets used by their study were collected based on a resting-state protocol considering only air stimulation. The characterised network revealed three active subnetworks distributed across sensory, limbic and frontal lobes. The results of their study indicated that the anterior insula and amygdala are two major hubs of the air processing network. Another study done by Zhou et al.^4^, also used a resting-state fMRI experiment and k-mean clustering to investigate the brain functional circuit underlying air processing. Their results showed that the primary olfactory cortex forms a distinct large functional network such that its pathways are spread across the whole brain. This network included the anterior olfactory nucleus, olfactory tubercle, frontal piriform cortex and temporal piriform cortex.

### Neural network evoked by odours

In contrast to previous studies which mainly focused on sniffing pure air, Ruser et al.^1^ employ fMRI to investigate the neural network underlying pleasant odour perception in healthy subjects. They used a block design paradigm including three olfactory conditions with different levels of pleasantness. The seed-to-voxel technique was used to measure functional connectivity in the brain. The results revealed that pleasant odour processing elicits functional connectivity among the posterior piriform cortex, insula, thalamus and right orbitofrontal cortex. In a similar study, Carlson et al.^12^ measured the functional brain network which was evoked by smelling odours conveying emotions. They used the Random Forest method to investigate differences between connectivity patterns evoked by pleasant and unpleasant odours. Their results revealed that pleasant and unpleasant odours have different effects on the functional connection between the insula and a subnetwork including the prefrontal cortex, amygdala, and hippocampus. In their study, three subnetworks including (i) amygdala, left DLPFC and left ACC, (ii) left piriform cortex, left insula and (iii) right piriform cortex and right PCC nodes were identified. The results also showed that unpleasant odours led to stronger functional connectivity among the identified subnetworks than pleasant odours. Reichert et al.^13^ performed a task-related fMRI experiment and independent component analysis (ICA) to investigate how emotional odours modulate memory function during image encoding and image retrieval tasks. Their fMRI experiment consisted of two sessions including an encoding task and a recognition task. At each task, a set of images were presented simultaneously with odours. There were three types of olfactory conditions comprising of congruent, incongruent and air. Their results indicated an stronger activity in the piriform cortex during the encoding task when congruent odours were presented. According to the ICA outcomes, it was revealed that the network constructed by the putamen, caudate and thalamus was modulated by incongruent odour during the retrieval task.

### Neural network evoked by chemosignals

In parallel with the research that explored brain connectivity in pure air and non-social odour processing conditions, a few studies have also been performed to characterise human neural networks activated by body odour. For example, Mishor et al.^14^ investigated whether sniffing hexadecanal (Hex) as a component of human body odour triggers aggression or not. They recruited both males and females to explore how a chemosignal (i.e Hex) affects gender-based social interactions. Their results showed that Hex significantly changed functional connectivity between the left angular gyrus and three regions including: (i) the right temporal gyrus extended to the middle temporal pole, (ii) the left amygdala extended to the left hippocampus, and (iii) the bilateral orbitofrontal cortex (OFC). It should be noted that the temporal gyrus is involved in social cue analysis while the amygdala and OFC are implicated in emotion processing and aggressive execution. The results of the between-subjects analysis revealed that smelling HEX triggered aggression in women while blocking it in men. In another study, Zhou et al.^9^ performed an fMRI experiment to identify brain network underlying human sexual chemosignals. They collected sweat from a group of healthy heterosexual male donors. Then, a group of healthy female subjects was asked to inhale and exhale the chemosignal stimuli after receiving a visual cue. They estimated functional connectivity by measuring the correlation of the fMRI time series. According to their results, the right orbitofrontal cortex and right fusiform cortex treat human chemosignals as social-emotional cues. However, functional connectivity investigation did not yield any significant correlation between these two regions. Additional exploration revealed a remarkable correlation between hemodynamic responses of the amygdala and piriform cortices. Moreover, their results reported a significant correlation between the right fusiform cortex and the right thalamus. Maier et al.^15^ also performed an fMRI study to investigate if oxytocin (OXT) can modulate the processing of chemosensignal of stress. They used chemosignals collected during sport and stressful conditions. Then the collected chemosignals were used in combination with oxytocin and placebo in a face emotion recognition task. They explored functional connectivity between anterior cingulate cortex (ACC) and FFA using psychophysiological interaction (PPI) analysis^16^. The results revealed that OXT reduces the effects of stress resulting from chemosignal by enhancing the functional connectivity between ACC and FFA. To our knowledge^9,15^ are the only studies that investigated functional connectivity during chemosignal processing. Although both studies provided good insights for researchers, the influences of chemosignals on many key regions in the olfactory system remained unclear due to including a very small number of brain areas in the analysis.

### Contribution

In this paper, we used the fMRI dataset collected by Zheng et al.^2^ to investigate the brain functional networks evoked by air, odours and chemosignals.

They assessed the effects of these olfactory stimuli on a 2-alternative-forced-choice task. Their fMRI analysis revealed distinct patterns of brain activity in response to different olfactory stimuli. The successful employment of social/non-social odours by Zheng et al.^2^ encouraged us to perform further analysis to investigate the functional neural circuits underlying these olfactory stimuli. It is important to note that Zheng et al. used emotional odours and chemosignals, meaning that those were either disgust or neutral. However, since the main goal of this study is to characterize the intrinsic (i.e. air), non-social (i.e. odour) and social (i.e. chemosignal) olfactory processing networks, the data was collapsed across the emotions for the network identification.

We proposed using graph theory to identify functional connectivity in the brain in response to each olfactory stimulus. Graph theory is a mathematical framework that can be used to model the pairwise communications between components of a network^17^. In particular, it allows the quantification of neuron communications during information transfer and processing. In this study, the graph theory uses the correlation of beta time series extracted from a number of key brain regions to characterize functional connectivity.

To identify the functional networks evoked by air, odour and chemosignal, the beta time series assosiated with each of these olfactory conditions were used. Identifying these networks would enable us to understand how the brain responds to pure air, social and non-social odours. Before characterizing the networks, and as a preliminary investigation, we conducted statistical analysis to assess the effects of experimental paradigm factors (i.e. social vs. non-social and disgust vs. neutral) on the measured correlations. We performed extensive analysis to assess the robustness and stability of the identified networks.Then the network’s features were evaluated to compare the roles of different brain regions in processing various olfactory stimuli.

## Materials and methods

### Participants

18 healthy female subjects who had normal olfactory function, no history of neurophysiological disorder and no history of taking psychotropic medications participated in this study. This sample size was chosen based on the findings of the previous studies performed in the same lab^18–20^ that supported a large effect size for the brain activity in many key regions such Amygdala $(Cohen's d= 0.94)$ and Piriform Cortex $(Cohen's d= 0.87)$. Subjects’ sense of smell was evaluated based on self-reported assessment of odour intensity and pleasantness rating during a lab visit. Individuals who showed abnormal olfactory performance or signs of nasal infection or allergies were excluded from the experiment. All subjects completed the informed consent to participate in the study which was approved by the University of Wisconsin–Madison Institutional Review Board. The experiment was performed in accordance with the approved guidelines and regulations. Two subjects who failed to complete the task were omitted from the study. The age range and average age of the remaining 16 subjects were 18–29 and 21 years respectively.

### stimuli

Five olfactory stimuli, including air, neutral odour, disgust odour, neutral chemosignal, and disgust chemosignal, which were produced by Zheng et al.^2^, were used in this paper.

The primary olfactory elicitors (i.e. odours) were produced in the chemistry lab using synthetic chemical compounds. Three neutral odourants including acetophenone $$(5\% l/l)$$, $$\alpha -$$pinene $$(10\%)$$, rose oxide/RO $$(5\%)$$ and three disgust odourants including trimethylaminuria$$/(5\%;$$ rotten fish), butyric acid $$(7.5\%;$$ rotten eggs), valeric acid $$(7.5\%;$$ sweaty socks) were generated. The concentration of the synthetic odourants was specified through systematic lab piloting to ensure comparable, moderate and effective intensity. The obtained disgust rating (1–10) for the disgust odours (mean(sd$$)=7.28(1.07))$$ and neutral odours (mean(sd$$)=2.57(1.96)$$ aligned with the intended emotion manipulation and showed a significant difference $$(p < 0.001)$$^2^.

The chemosignals (i.e. sweats) were collected from 14 healthy male donors. Their mean age was 19.8 years and they were heterosexual and nonsmokers. The sweat donors adhered to strict dietary and behavioural restrictions including avoiding odorous foods, alcohol, smoking, deodorants, scented products, sexual activity, and strenuous exercise for two days before and on the day of the sweat donation. This ensured minimal extraneous odours in their sweat samples^3^. To induce emotions, the donors watched disgusting and neutral video clips during sweat collection. The donor’s sweats were collected using absorbent compressed pads under their armpits. The sweat pads showed a significant increase in weight after watching the video (before :  mean(sd$$)=4.95(0.24)$$g, after  :  mean(sd)$$=5.26(0.55)$$g$$; t=2.98, p = 0.005)$$. The sweat pads’ weight was not different between the two emotional conditions before $$(p=0.16)$$ and after $$(p=0.31)$$ watching video clips. The subjective disgust ratings (1–10) for the disgust and neutral sweats were mean(sd$$)=2.44(2.18)$$ and mean(sd$$)=1.48(1.67)$$ respectively showed that both were perceived as equally neutral $$(p = 0.14)$$, reflecting the inherent nature of chemosignals and aligning with previous findings^21,22^. More details about the stimuli are available in^2^.

### Experimental paradigm

**Fig. 1:**
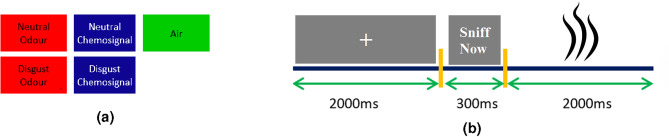
(**a**) The utilised olfactory stimuli. (**b**) The time-line of one trial: any of the olfactory stimuli is presented to the subject after a 2 s grey fixation and a 300 ms cue.

The experimental paradigm included five olfactory conditions each including 12 trials. Figure 1a,b depict the olfactory conditions and the timeline of one trial respectively. Each trial is started with a grey fixation crosshair displayed on the screen for 2 s. Then a visual cue reading “sniff now” appears on the screen. The visual cue is followed by a 2 s chemosignal/odour/air delivery. The olfactory stimuli (i.e. odours, chemosignals and air) were delivered using an MRI-compatible sixteen-channel computer-controlled olfactometer (airflow set at 1.5 L/min) at room temperature. The stimuli were also pseudo-randomized to avoid repetition over the trials.

### fMRI acquisition and preprocessing

Details of fMRI scanning parameters can be found in an earlier published study^2^. We replicated the same preprocessing pipeline as^2,23^ using the statistical parametric mapping (SPM) toolbox^24^ for reducing noises and correcting the artifacts.

The preprocessing pipeline included removing six dummy volumes from the beginning of each session, slice time correction, spatial realignment followed by field map correction, fMRI and sMRI coregistration, segmentation of the coregistered structural MRI into different brain tissues for generating a template in MNI space, normalisation of fMRI volumes using generated template and smoothing with a 6-mm full-width half maximum Gaussian kernel.

The preprocessed fMRI images were then used to extract beta time series associated with each olfactory condition.

### Functional connectivity analysis

Our aim in the present study is to explore the functional networks underlying air, odour and chemosignal processing. We therefore conducted functional connectivity analysis by performing four main stages including (i) extracting beta series from a set of regions of interest (ROIs) and estimating the correlation matrices using the BASCO toolbox^25^, (ii) constructing networks using estimated correlation matrices, (iii) characterising the topology of networks using Gephi software^26^, and (iv) measuring and comparing the metrics of the identified networks.

(i) Estimating beta series and correlation matrices: beta series correlation is appropriately designed to characterise functional inter-regional connectivity in event-related fMRI experiments^27^. In this study, we used the general linear model (GLM) approach integrated into the BASCO toolbox^25^ to measure the brain activity evoked by each olfactory stimulus at each trial. Six head motion parameters estimated during preprocessing were also added to the GLM design matrix for regressing out the nuisance effects of head movement. The estimated beta time series associated with each olfactory condition were then used to measure inter-regional correlations for each olfactory condition and each individual subject. The measured correlation value between the two regions’ beta series reflects their functional connectivity level in a particular condition. In this study, we measured beta series correlation among a set of 29 brain regions of interest that have previously been reported to be active during olfaction and chemosignal perception^5,15^. The selected ROIs can be categorised into several groups based on their functionality, including primary and secondary olfactory cortices, the limbic system, decision-making regions, and the social interaction processing areas. In the following, the selected ROIs and their associated groups are introduced. The first group involves the anterior and posterior piriform cortex(APC, PPC), amygdala(AMY), entorhineal cortex (ENT), olfactory OFC (Oolf) and olfactory tubercle (OTB) which are key primary olfactory regions that receive signals directly from the olfactory bulb^28^. Dorsal anterior and posterior thalamus (THLda and THLdp), ventral anterior and posterior thalamus (THLva and THLvp), anterior, posterior, dorsal and ventral insula (INSa, INSp, INSd, INSv), anterior and posterior hippocampus (aHIP, pHIP), lateral olfactory OFC (Oolfl), nucleus accumbens (NAcc), and hypothalamus (HYP) are the regions associated with the secondary olfactory cortex^28^. Some of these areas such as AMY, HYP, thalamus and insula are key regions involved in emotion processing and limbic system^29^. Areas such as middle and posterior medial OFC (mOFC, pmOFC), anterior, posterior and medial lateral OFC (alOFC, plOFC, mlOFC), anterior and central OFC (aOFC, cOFC), posterior middle OFC (mpOFC) and anterior AFC-OFC (aAOFC) are involved in decision making tasks^30^. It has been observed that body odour triggers a strong response in the fusiform face area^2,16^. Therefore, FFA was also considered as one of the regions of interest in our study. FFA is located in the visual cortex and is recruited for processing faces includes emotional expressions. Since the face communicates fundamental social information, FFA can be considered a key node of the social group. In addition, activity in the FFA during body odour smelling suggests that the brain processes body odour as a social stimulus^8^. We informed BASCO by the anatomical masks generated by^5^ to enable it to extract beta series from ROIs. More details about brain parcellation and the creation of the anatomical masks are available in^5^. FFA was not included in^5^ analysis, therefore we generated an anatomical mask for this region using the WFU$\_$PickAtlas toolbox^31^. In order to measure correlation matrices for each individual subject and each olfactory condition, we estimated the Pearson correlation for each pair of condition-specific beta series extracted from regions of interest. As a result of having 29 ROIs, a $29\times 29$ correlation matrix was obtained for each particular condition. Since the experimental paradigm consisted of 5 olfactory conditions, 5 correlation matrices were calculated for each individual subject respectively. All the estimated weights were then Fisher-Z transformed to be suitable for further analysis^32^. It should be noted that for network construction, we ignored the effects of emotions by collapsing data across this factor and looked only at the networks underlying three olfactory stimuli (i.e. air, odour and chemosignal).
(ii) Network construction: Three key steps were conducted to construct the network for each olfactory condition. The first step was averaging the measured correlation matrices for each condition across subjects and emotions. This led to obtaining one group correlation matrix for air condition, one group correlation matrix for odour condition and one group correlation matrix for chemosignal condition. The second step was computing the weighted adjacency matrices from the group correlation matrices by removing weak or non-significant connections. The last step was applying the modularity optimization algorithm proposed by^33^ on weighted adjacency matrices to partition the connections into modules. In our study, the weighted adjacency matrices, which contain significant connections (i.e. correlations), were identified by applying appropriate thresholds on the group correlation matrices. Without a proper threshold, the network may become either too sparse (missing important connections) or too dense (including noisy, irrelevant connections). To find an optimal threshold for each group correlation matrix, we examined the variation in the modularity index which measures the network robustness by changing the threshold values. Modularity index (Q), whose value lies in the range $[-0.5$ 1], illustrates the structure of a graph (i.e. network) by measuring the density of connections within a subnetwork^34^. The value of this index indicates the stability of a network’s community structure by measuring its resistance to perturbations. We gradually changed the threshold value to obtain a set of weighted adjacency matrices containing between $5\%$ and $50\%$ suprathreshold connections^35^. Then each weighted adjacency matrix was submitted to the Louvain algorithm^36^ to conduct modularity analysis. The main objective of modularity analysis is to construct a network based on the weighted adjacency matrix by finding the best deviation on connections into subnetworks or modules. The best deviation is characterised by a maximum number of intramodule connections while keeping intermodule connections at the minimum possible level. The measured modularity index associated with each threshold indicates how much the network generated by suprathreshold connections satisfies this criterion. The recommended score for the modularity index is 0.3 indicating strong evidence of existing subnetworks^37^. Therefore, a threshold that leads to obtaining a modularity index of 0.3 can be considered as an optimal threshold. After determining the optimal threshold, modularity maximization procedure was conducted to identify subnetworks at the optimal threshold, using the algorithm proposed by Schuetz et al.^38^.
(iii) Network topology presentation: after identifying the structure of the networks, we used Gephi software^26^ to draw a schematic representation of subnetwork organisations and their connections. Force Atlas was used as the layout to draw the network topology. In addition, the modularity class which was assigned to each module by the Louvian technique was used to partition the nodes.
(iv) Graph metrics: we then characterised and quantified global and local network features. Globally, we examined metrics including “global efficiency”, “clustering coefficient”, “modularity index” and “average weighted degree”. Global efficiency refers to network integration and global communication. This index is computed by averaging the inverse of shortest path lengths across all pairs of nodes^39^. The shortest path length between two nodes in a graph is the minimum number of graph edges that should be travelled to get from one node to another. We also measured the average path length which is the average of the shortest path lengths across all pairs of nodes^39^. Both the average path length and global efficiency indicate how easily information is transferred across the network^40^. In a small world network, the global efficiency should be close to that of a random network that is constructed by plenty of random connections. The clustering coefficient quantifies the degree to which nodes in a graph tend to cluster together. The global clustering coefficient of a graph is the average of clustering coefficients across all network nodes^40^. Generally, in the real world, nodes tend to be knitted tightly and create a dense group which is characterised by a high density of mutual interconnections. In a small world network, the clustering coefficient should be higher than in a random network because the random connections do not tend to generate clusters. The average weighted degree is another global index that was measured in this study. A graph is comprised of nodes and their connecting edges. The strength of each graph’s edge is weighted by the correlation between nodes that are connected by this edge. The average weighted degree is measured by the summation of all the weights given to edges divided by the number of nodes. This global index should be higher in small world networks than random networks.

In addition to global features, a set of local features including “hub score”, “degree”, “betweenness” and “closeness” were also computed. These indices measure how efficiently the networks exchange information locally. Hub score is a metric that quantifies the importance of a node’s edges for facilitating network traffic^41^. Node degree is the number of direct connections between a given node and any other nodes in the network^40^. Higher values of this metric indicate that many nodes are attached to the given node. Betweenness indicates how much a node is in-between the other nodes. This metric measures the number of shortest paths that are travel through a given node divided by the total number of the existing shortest paths in the graph^42^. A high estimated betweenness for a specific node indicates appearing in many signal transfers. Closeness captures the centrality of a node in the graph by the inverse reciprocal of the average of shortest path lengths between the given node and others^42^. Small closeness centrality indicates that the node is less connected to other nodes in the graph and is not in the centre of the network to transfer information.

### Additional analyses

As a complementary analysis, the contribution of each node to network efficiency was assessed by targeted node deletion. For this purpose, we removed each node iteratively and then the percentage of global efficiency and clustering coefficient reduction were calculated^39^. We also measured participation coefficients to determine whether a node with a high hub score was a connector or provincial hub. The participation coefficient indicates the diversity of module connections of a given node^43^. The nodes with high hub scores and high participation coefficients are called “connector” hubs. In contrast, nodes with high hub scores and low participation coefficients are called “provincial” hubs^43^. The edges of the provincial hubs are restricted to its module while the edges of connector hubs are widely distributed among all network modules.

## Results

### Effects of sources and emotions on connections

This section presents the results of an investigation into how the sources (i.e. social/non-social) and emotions (i.e. disgust/neutral) of the utilized odours influenced the strength of connections. A 2-way ANOVA was used here to compare the odour and chemosignal correlation matrices before averaging them across the emotions. The results of statistical analysis were corrected for multiple comparisons by the Tukey-Kramer method at a significance level $p = 0.05$. Table 1 represents the connections whose strength was modulated by the experimental paradigm factors. According to the results, AMY-INSa and aHIP-pmOFC connections were modulated by both factors and their interaction. aAOFC-FFA was modulated by both sources and emotions while no modulation due to interaction was observed. The results indicated that AMY-cOFC, pHIP-INSp were modulated by the source of odours. pHIP-plOFC, INSd-INSv and Oolfl-mOFC were connections whose strength were modulated by the interaction of sources and emotions. Moreover, the pHIP-pOFC connection was also modulated by emotions delivered by odours.

**Table 1:** Results obtained by 2-way ANOVA to investigate the effects of sources and emotions of olfactory stimuli on networks’ connections. Each row represents the statistical significance of a modulated connection in the form of $({F,p,\omega ^{2}})$. where *p* is the corrected $p_{value}$ and $\omega ^2$ is the effect size.

Connection	Effect of sources (odour vs. chemosignal)	Effect of emotions (disgust vs. neutral)	Interaction of sources and emotions
AMY $\rightarrow$ INSa	(6.20,0.0160,0.25)	(4.04,0.04,0.18)	(9.52,0.0032,0.36)
AMY $\rightarrow$ cOFC	(4.74,0.034,0.21)	–	–
aHIP $\rightarrow$ pmOFC	(10.42,0.0027,0.38)	(9.86,0.0027,0.37)	(9.86,0.0027,0.37)
pHIP $\rightarrow$ INSp	(7.72,0.0075,0.30)	–	–
pHIP $\rightarrow$ plOFC	–	(4.98 , 0.029,0.22)	(6.91,0.011,0.29)
INSd $\rightarrow$ INSv	–	–	(4.78,0.033,0.21)
Oolfl $\rightarrow$ mOFC	–	–	(4.51,0.038,0.20)
aAOFC $\rightarrow$ FFA	(6.10, 0.0167,0.25)	(4.50,0.0386,0.20)	–

**Fig. 2:**
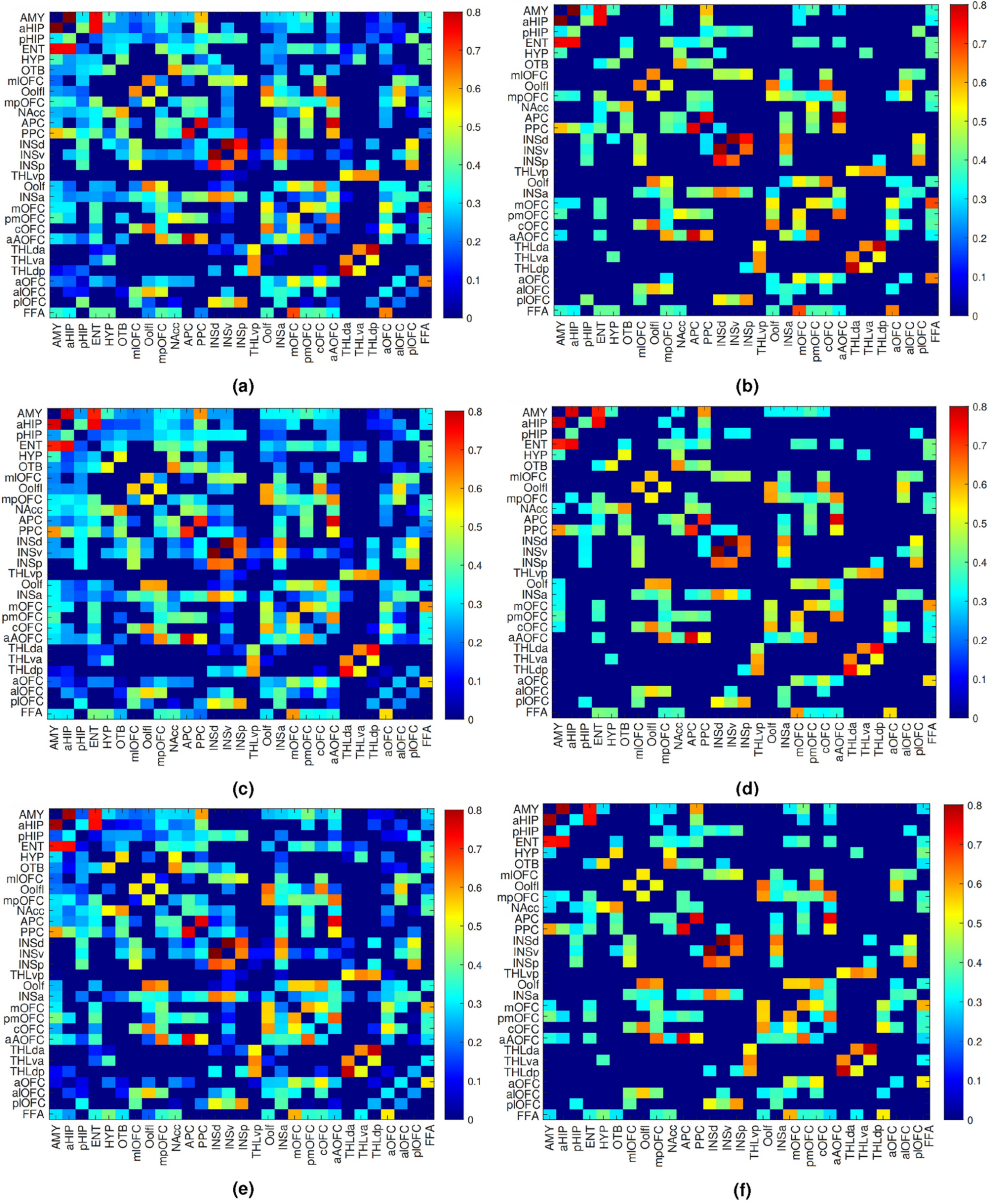
The average correlation weights of (**a**) air, (**c**) odour, and (**e**) chemosignal conditions. The suprathreshold correlation weights for (**b**) air, (**d**) odour and (**f**) chemosignal conditions. The abbreviated name of the brain regions is written next to each matrix row and column.

### Intrinsic, odour, and chemosignal connectivity matrices

Figure 2a,c,e shows the correlation matrices obtained by averaging across subjects and emotions at air, odour and chemosignal conditions. As it was mentioned in the method section, the emotional conditions were merged by averaging so that emotion was not considered as a distinct factor in the network characterisation analysis. Each element of the correlation matrix represents the measured correlation weight between two nodes of the network. Figure 2b,d,f shows the weighted adjacency matrices obtained by applying thresholds on the group correlation matrices.

**Fig. 3:**
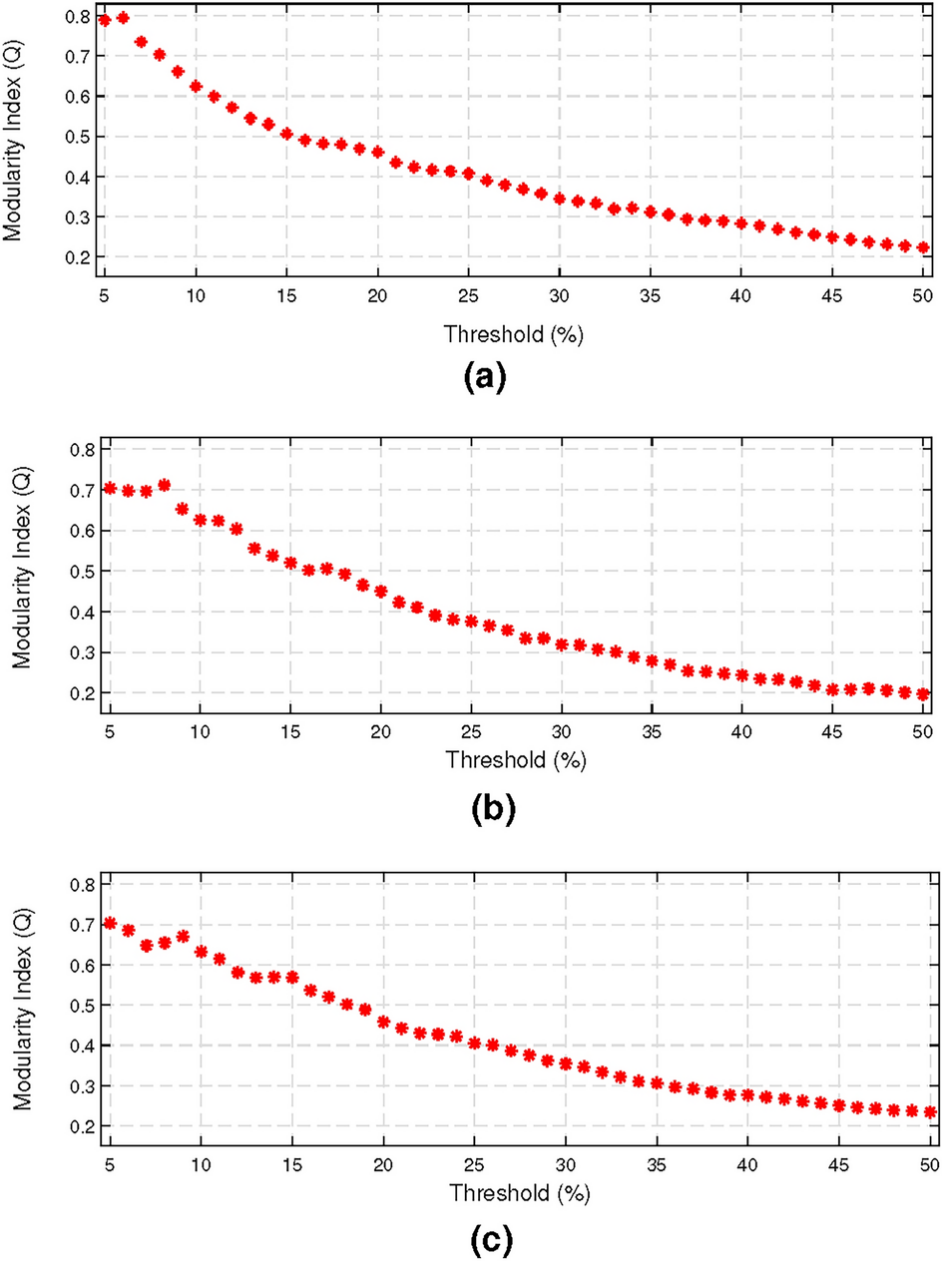
Variation of modularity index across different thresholds for (**a**) air (**b**) odour and (**c**) chemosignal networks. According to the results, in air, odour and chemosignal conditions, the modularity index reached to 0.3 when $35\%$, $32\%$, and $35\%$ of the strongest connections were used to construct the network respectively.

We estimated the optimal thresholds using the procedure explained in the Network construction section. According to that procedure, we applied different thresholds on correlation matrices and measured the modularity index for the obtained weighted adjacency matrices. Figure  3 represents the estimated modularity indices obtained for three olfactory conditions at different threshold values. Threshold that led to obtaining modularity index of 0.3 were chosen as the ptimal thresholds^37^. As seen in Fig. 3, the optimal thresholds were $35\%$, $32\%$ and $35\%$ for air, odour and chemosignal conditions respectively.

### Network organisation and characterisation

**Fig. 4:**
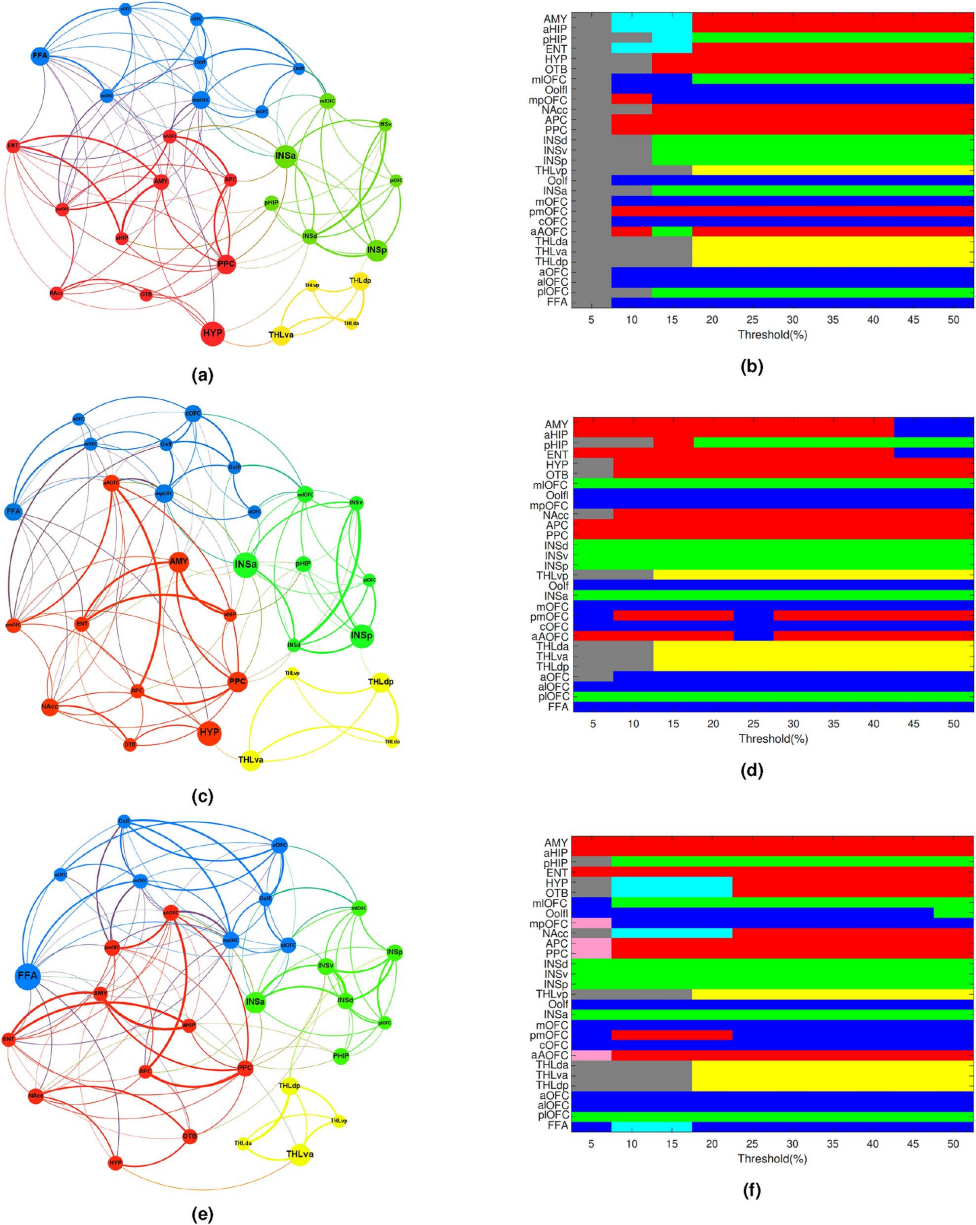
Topology of the networks characterised for (**a**) air (**c**) odour and (**e**) chemosignal conditions. These are the most robust network typologies that were obtained with $35\%$, $32\%$ and $35\%$ strongest connections in air, odour and chemosignal conditions respectively. Also shown is the variation of module assignment across different thresholds for (**b**) air, (**d**) odour and (**f**) chemosignal conditions. Each row corresponds to one node and each column corresponds to one threshold applied to the correlation matrix. Colours indicate how nodes were assigned to different modules at different thresholds. Disconnected nodes were shown by grey colour.

Figure 4a,c,e show the topology of the networks that were characterised using the suprathreshold correlation coefficients, available in the weighted adjacency matrices, for three olfactory conditions. The characterised networks comprised of four subnetworks (i.e. modules) that are presented using four colours in Fig.  4a,c,e. The sensory module that was shown by red colour comprised of key primary olfactory regions including APC, PPC, OTB and ENT. Some regions associated with the secondary olfactory cortex including NAcc, aHIP, pmOFC and aAOFC were also connected to this module. Observing such a large cluster that provides functional connectivity between the primary olfactory cortex and two sub-regions of OFC (i.e. pmOFC and aAOFC) could explain the strong activity of OFC in olfactory tasks^2^. OFC is a frontal brain region that is mainly involved in object recognition, face processing, and decision-making tasks and also plays a crucial role in behaviour control^44^. In addition, AMY and HYP which are often active in emotion processing were connected to this module too. The second module is presented in blue in the Fig. 4a,c,e. This module revealed an extensive area of connectivity among various parts of OFC including olfactory, anterior, central, lateral, anterior-lateral, posterior-medial and middle-medial. The connection between FFA and most parts of OFC was also established in this module. Another module that was characterised by our analysis consisted of four parts of the insula, two lateral parts of OFC (median and posterior) and the posterior hippocampus. This module is distinguished by its green colour. The insula, which is located in the secondary olfactory cortex, is involved in a wide range of functions including sensory and cognitive tasks^45^. The smallest identified module, shown by yellow colour, revealed an intra-modular connection among four thalamus parcels.

In another analysis, we investigated whether this modularity output was dependent on connectivity density. We explored the modules across a wide range of connectivity densities (5–50%). As illustrated in Fig. 4b,d,f, the modularity output was reasonably consistent. It was particularly consistent for all conditions and at $>25\%$ density. Therefore, modular analysis at the threshold $(35\%$ and $32\%$ density) could be considered as reliable. We also assessed the small-worldness of the networks by measuring the global features of the identified networks and comparing them with the features of a random network. The random networks were generated for each condition by 10, 000 permutations of the correlation coefficients associated with that condition. The estimated global features of the identified and random networks are shown in Table 2. Based on the obtained results, all the estimated olfactory networks indicated a high degree of small-worldness with comparable global efficiency and higher clustering coefficients to the random network. In addition, comparing the average path length of the estimated networks and the associated random networks indicated simplification of information transfer in the identified olfactory networks. The last measured global feature revealed that the estimated networks were characterised by higher average weighted degrees rather than random networks. The high average weighted degree indicated that the nodes of these networks were visited many times supporting the hypothesis that the estimated olfactory networks were not random.

**Table 2:** Estimated global features for the characterised and random networks.

	Global efficiency		Clustering coefficient		Average path length		Average weighted degree	
	Estimated	Random	Estimated	Random	Estimated	Random	Estimated	Random
Air	0.268	0.280	0.310	0.289	2.084	2.096	3.771	3.750
Odour	0.257	0.273	0.285	0.254	2.128	2.131	3.466	3.454
Chemosignal	0.256	0.271	0.288	0.269	2.019	1.990	3.670	3.662

**Fig. 5:**
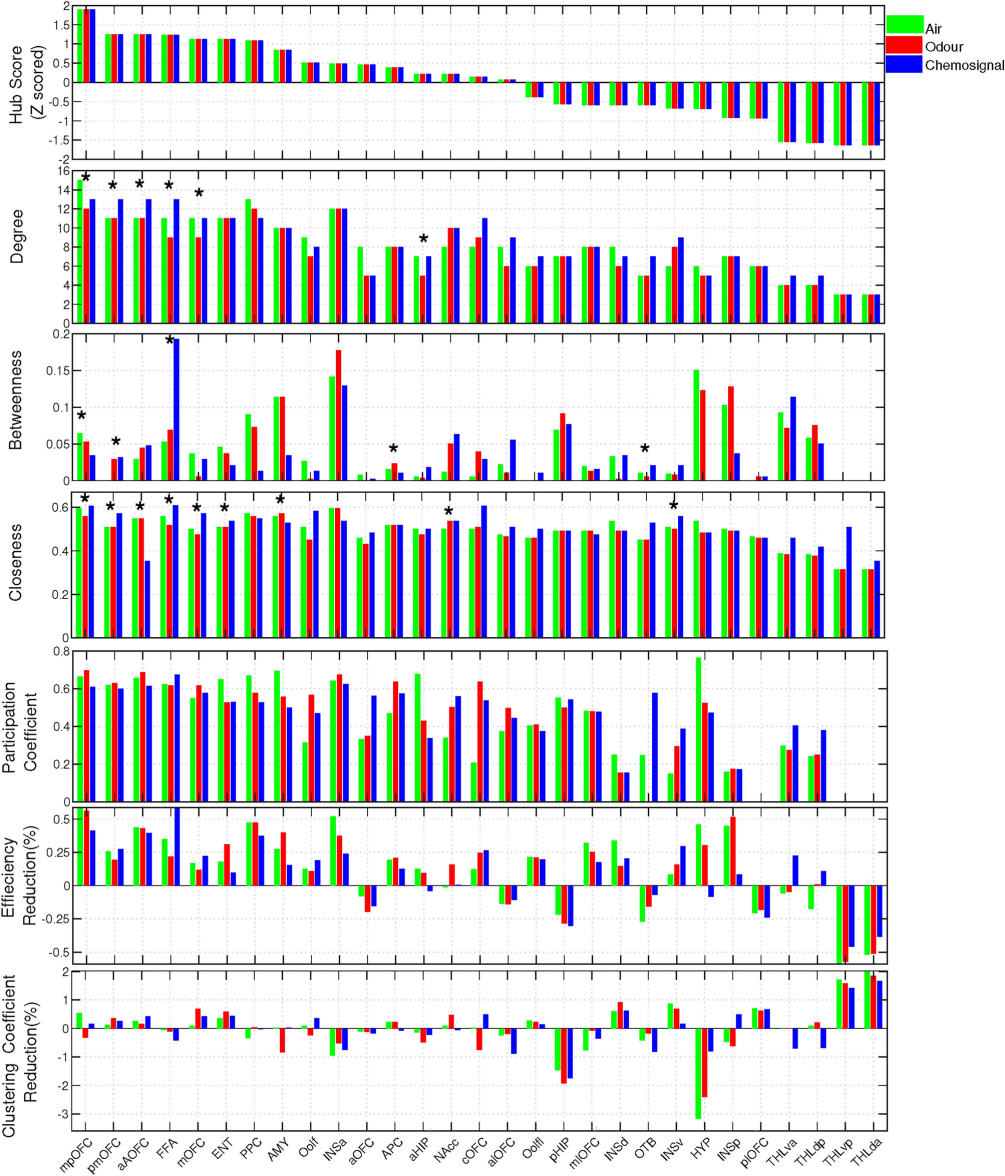
Local network features and evaluation of network global features against local attacks. The nodes that had different centrality indices at each network are distinguished by $*$ in the figure.

After evaluating the global features, we measured the local features to study subnetwork organisations and node roles precisely. Figure 5 represents the measured local features. The first measured local feature was the hub score. In Fig. 5, the order of nodes was sorted based on the measured hub scores from the highest to lowest value. According to the results mpOFC obtained the highest hub score among all nodes. The remaining nodes whose Z-scored hub indices were higher than zero can be divided into three groups based on the value of the hub score. The first group included pmOFC, aAOFC, FFA, mOFC, ENT and PPC with hub scores between one and two. The second group included AMY, Oolf and INSa with hub scores between 0.5 and one. The third group included aOFC, APC, aHIP, NAcc, cOFC and alOFC with hub scores between zero and 0.5. In addition to the hub score, the degree, betweenness and closedness of each node were also measured as indices of centrality. We performed the analysis of variance (ANOVA) at this stage for statistical validation of the connectivity pattern. The results of ANOVA were then corrected for multiple comparisons using the tukey-kramer method and significance level $p=0.05$. Table  3 represents the corrected results associated with each centrality index. The measured degrees are shown in the second top row of Fig. 5. As seen from the figure, the nodes with high hub scores also had high degrees. The obtained results revealed that mpOFC, PPc and INSa are the first three nodes with the highest degree respectively in the air and odour networks. In the chemosignal network, four nodes, including mpOFC, pmOFC, aAOFC and mOFC had the highest degrees with the same value among all nodes. The statistical comparison revealed six significant differences among the degrees measured for mpOFC, pmOFC, aAOFC, FFA, mOFC and aHIP. The results of multiple comparisons with the corrected *p* values were presented in Table  3.

**Table 3:** ANOVA with post-hoc multiple pairwise statistical comparison. The table is divided into three sections, each section is associated with one pairwise comparison. Each row indicates the node that had significantly different features (i.e. Degree, Betweenness and Closeness) in the compared networks. The reported results are in the form of (*F*,*p*,$\omega ^2$) that *p* is the corrected $p_{value}$ and $\omega ^2$ is the effect size).

	Degree	Betweenness	Closeness
	Air vs. Odour		
mpOFC	(4.96, 0.007, 0.22)	–	(9.52, 0.00027, 0.36)
pmOFC	-	(6.52, 0.0084, 0.27)	–
FFA	–	–	(18.297, 0.04, 0.52)
mOFC	(6.54, 0.061517, 0.27)		
AMY	–	–	(8.05, 0.0026, 0.32)
aOFC	–	–	
APC	–	(3.54, 0.045, 0.16)	–
aHIP	(4.70, 0.031, 0.21)		
NAcc	–	–	(3.97, 0.041, 0.18)
	Air vs. Chemosignal		
pmOFC	$(17.03,5.7087{\cdot } 10^{-5},0.52)$	(6.52, 0.008, 0.27)	$(28.15,4.693{\cdot } 10^{-7},0.63)$
aAOFC	(4.54, 0.029, 0.21)	–	(6.55, 0.028, 0.27)
FFA	$(25.68,4.902{\cdot } 10^{-8},0.61)$	(6.88, 0.003, 0.29)	$(18.297,8.6275{\cdot } 10^{-7},0.52)$
mOFC	–	–	(8.97, 0.00039, 0.33)
ENT	–	–	$(20.27,4.5759{\cdot } 10^{-6},0.57)$
NAcc	–	–	(3.97, 0.045, 0.18)
OTB	–	(6.694, 0.016, 0.26)	
INSv	–	–	(5.64, 0.004, 0.24)
HYP	–	–	(8.75, 0.0014, 0.33)
	Odour vs. Chemosignal		
mpOFC	–	(3.89, 0.036, 0.18)	(9.52, 0.017, 0.36)
pmOFC	$(17.03,1.0052{\cdot } 10^{-5},0.52)$		$(28.15,6.8799{\cdot } 10^{-8},0.63)$
aAOFC	(4.54, 0.035, 0.21)		
FFA	$(25.68,1.389{\cdot } 10^{-5},0.61)$	(6.88, 0.012, 0.29)	(18.297, 0.0028, 0.52)
mOFC	(6.54, 0.002, 0.27)	–	(8.97, 0.024, 0.33)
ENT	–	–	$(20.27,5.0731{\cdot } 10^{-6},0.57)$
AMY	–	–	(8.05, 0.003, 0.32)

The measured betweennesses showed that INSa has the highest betweenness centrality in air and odour networks, while FFA has the highest betweenness in the chemosignal network. The analysis of variance for betweenness centrality revealed some significant differences between conditions. According to the results obtained by multiple comparisons pmOFC and APC had different betweenness in air and odour networks. Comparing air and chemosignal networks indicated that pmOFC, FFA and OTB have different betweenness in these conditions. In addition, two nodes including mpOFC and FFA had different betweenness in odour and chemosignal.

Calculating the closeness of all nodes revealed that INSa and mpOFC have the highest closeness centrality in the air network. In the chemosignal and odour network, the highest closeness was obtained by FFA and INSa respectively. ANOVA detected many nodes with different closeness values across the olfactory conditions’ network (Table  3).

We also measured the participation coefficients to investigate whether the nodes that achieved high hub scores were connector hubs or provincial hubs. According to the results (Fig.  5) AMY, aHIP and PPC achieved the highest three participation coefficients in the air network. In the network characterised for odour condition, mpOFC, aAOFC and INSa achieved the highest three participation coefficients. In the chemosignal network, the highest three participation coefficients were obtained for FFA, INSa and aAOFC.

As an extended study, we assessed the stability of the networks against local attacks by removing nodes iteratively and measuring the reduction of global efficiency and clustering coefficient. The results of this experiment were presented in the last two rows of Fig. 5. Based on the results, removing the hubs (i.e. aAOFC, FFA, PPC, AMY, mpOFC and INSa) severely reduces the global efficiency and clustering coefficient of the networks.

## Discussion and conclusion

In this study, we used event-related fMRI and graph theory to investigate the functional brain networks underlying air, odour and body odour (chemosignal) processing. Our results provided a good insight into how the brain responds to social and non-social odours. Although the experimental manipulation of the odourants (i.e. emotions) was not considered in network analysis, the results revealed significant differences in various key features of the identified odour and body odour networks. Not only does this observation support the existence of chemosignal in the body odours^46^, but it also is aligned with the literature that claimed different brain networks underling non-social and social odour processing^8^. One of the key findings of our results is detecting the FFA as one of the connector hubs in the chemosignal network due to having a high hub score and the highest participation coefficient compared with odour and air networks. Although the FFA is typically considered a visual area, this finding aligns with previous studies suggesting that it processes human body odors as social cues^2,9,15^. Additionally, our results revealed that all three FFA centrality features differ significantly between the odour/air and chemosignal conditions, further supporting the hypothesis of employing social areas by body odour network 5. The results also indicated the major role of the posterior part of OFC (i.e. mpOFC and pmOFC) in the identified networks by demonstrating different centrality features reported in 3. OFC is also a key region involved in social cue processing^47,48^. According to the literature, individuals with orbitofrontal cortex damage often exhibit impaired social judgment^49^. Apart from this, no visual stimuli (e.g., faces or social images) were included in our analysis due to using the beta time series associated with olfactory stimuli. This arrangement, combined with the observed features in the FFA, mpOFC, and pmOFC, led us to speculate that these differences correspond to the social content of the olfactory stimuli.

The intrinsic olfactory processing network characterised in our study showed strong consistency with the air network identified by Arnold et al.^5^. In^5^, a robust network was identified for the intrinsic human olfactory system based on (i) the large human connectome project (HCP)^50^ and (ii) an independent resting-state fMRI dataset. Not only both studies observed similar strong correlations, but also the pattern of the air correlation matrix obtained by our analysis is consistent with the correlation matrices reported by^5^. In addition, AMY was detected as a connector in the air network by both studies. Our results indicated that the thalamus parcels have the smallest hub score in the characterised networks. This is consistent with the findings of^5^ that indicated the small contribution of the thalamus in the olfactory network.

The global features of the characterised networks, reported in table  2, support the small-worldness of olfactory processing through balancing local specialization (high clustering) and efficient global communication (high global efficiency)^39,51^. Moreover, the modular organisation of the identified networks depicted in Fig. 4a,c,e and their robustness across the thresholds illustrated in Fig.  4b,d,f supports efficient local processing of olfactory stimuli within modules while maintaining quick global communication between them.

While this study provides valuable insights into the olfactory processing network, several limitations should be acknowledged. First, the sample size was relatively small and included only female participants, introducing potential gender bias and limiting the generalizability of the findings. Another limitation is the thresholding methodology used to construct the functional networks, while robust, may have influenced the modularity outcomes, as different thresholds can alter network structure and connectivity patterns. Additionally, the study focused primarily on functional connectivity without examining behavioral correlations, which could have strengthened the interpretation of how olfactory stimuli influence perception and behavior. This shortcoming can be addressed in another study by estimating the dynamic brain networks (i.e. effective connectivity) underpinning processing different olfactory stimuli. Effective connectivity^52^ would enable us to assess how the brain connections are dynamically modulated by the experimental paradigm factors (i.e., both source and emotion). Finally, validating the role of the fusiform face area and some parts of OFC in processing social odours needs further investigation. Exploring the effective connectivity would also help to further clarify whether the strong activation of these two nodes is due to direct olfactory processing or the integration of olfactory cues with social signals.

## Data Availability

The dataset generated and analysed during the current study is available from the corresponding author on reasonable request. The generated code can be obtained from the corresponding author upon request without any additional restrictions.
